# Studies of trypanosomiasis in the Luangwa valley, north-eastern Zambia

**DOI:** 10.1186/s13071-015-1112-y

**Published:** 2015-09-30

**Authors:** Dusit Laohasinnarong, Yasuhuki Goto, Masahito Asada, Ryo Nakao, Kyoko Hayashida, Kiichi Kajino, Shin-ichiro Kawazu, Chihiro Sugimoto, Noboru Inoue, Boniface Namangala

**Affiliations:** O.I.E. Reference Laboratory on Surra, National Research Center for Protozoan Diseases, Obihiro University of Agriculture and Veterinary Medicine, Inada-cho, Obihiro, Hokkaido 080-8555 Japan; Clinical Sciences and Public Health Department, Faculty of Veterinary Science, Mahidol University, 999 Phuttamonthon 4 Road, Salaya, Phuttamonthon, Nakhon Pathom, 73170 Thailand; Department of Molecular Immunology, Graduate School of Agricultural and Life Sciences, The University of Tokyo, Bunkyo-ku, Tokyo 113-8657 Japan; Research Center for Zoonosis Control, Hokkaido University, Sapporo, Hokkaido 060-0818 Japan; Department of Paraclinical Studies, School of Veterinary Medicine, University of Zambia, P.O. Box 32379 Lusaka, Zambia

**Keywords:** AAT, Glossina, LAMP, PCR, Trypanosomiasis, Zambia

## Abstract

**Background:**

The present study, conducted in Zambia’s Luangwa valley where both animal African trypanosomiasis (AAT) and human African trypanosomiasis (HAT) are endemic, combined the use of microscopy and molecular techniques to determine the presence of trypanosome species in cattle, goats and tsetse flies.

**Methods:**

This study was conducted between 2008 and 2010 in Petauke, Chama and Isoka districts, north-eastern Zambia. A total of 243 cattle, 36 goats and 546 tsetse flies, were examined for presence of trypanosome species using microscopy, PCR and loop-mediated isothermal amplification (LAMP).

**Results:**

There was poor agreement among the test methods used for detection of trypanosomes species in animal blood and tsetse flies. Trypanosomes were observed in 6.1 % (95 % CI: 3.3-8.9 %) of the animals sampled by microscopy, 7.5 % (95 % CI: 4.4–10.6 %) by PCR and 18.6 % (95 % CI: 13.6–23.6 %) by PFR-LAMP. PFR-LAMP was more sensitive for detecting *Trypanozoon* than KIN-PCR. The highest occurrence of AAT was recorded in cattle from Petauke (58.7 %, 95 % CI: 44.7–72.7 %) while the lowest was from Isoka (5.4 %, 95 % CI: 0.8–10.0 %). Infection of both cattle and goats with *Trypanosoma congolense* and *T. vivax* was associated with clinical AAT.

**Conclusion:**

When selecting molecular techniques for AAT surveillance in endemic regions, the KIN-PCR and species-specific PCR may be recommended for screening animal or tsetse fly samples for *T. congolense* and *T. vivax*, respectively. On the other hand, species-specific PCR and/or LAMP might be of greater value in the screening of animal and human body fluids as well as tsetse fly samples for *Trypanozoon*.

## Background

Animal African trypanosomiasis (AAT) is one of the most important arthropod-borne diseases of cattle and other domestic animals in sub-Saharan Africa (SSA). It has a major impact on livestock productivity in SSA, with 50 million cattle and 70 million small ruminants at risk, costing up to US$5 billion annually [1]. *Trypanosoma congolense* and *T. vivax*, mainly transmitted by tsetse flies (*Glossina* spp.), are the major causes of AAT in domestic ruminants [2–4]. The disease is usually chronic and debilitating and characterized by intermittent fever, progressive anaemia, emaciation, lymphadenopathy and may lead to death if untreated [4]. Anaemia is the most pathogenic consequence of AAT [5]. *Trypanosoma brucei* subspecies (*T. b. brucei* s.l) are considered to have low pathogenicity to domestic ruminants. Cattle have been documented to serve as reservoirs of *T. b. rhodesiense*, the aetiological agent of human African trypanosomiasis (HAT), in Uganda [6, 7], Kenya [8] and Tanzania [9–11]. HAT in Zambia is thought to be endemic in the old foci (Luangwa, Zambezi and Kafue river valleys), with sporadic cases being reported [12–15]. In Zambia, HAT is transmitted by tsetse flies species of *Glossina morsitans morsitans, Glossina pallidipes, Glossina morsitans centralis* and *Glossina brevipalpis* [16], with several domestic and wild animals acting as reservoir hosts [17–19] .

Field diagnosis of AAT is complex since the clinical signs are not pathognomonic. Visualization of parasites in body fluids by microscopy is currently the gold standard of AAT and/or HAT diagnosis in endemic regions [20, 21]. Animals often exhibit a low but persistent parasitamia which fluctuates below the levels of microscopic detection [5, 21]. Over the past two decades, polymerase chain reaction (PCR) and Loop-mediated isothermal amplification (LAMP) techniques have been applied to improve the sensitivity and accuracy of AAT and HAT diagnosis [22–25] that are often applied in large-scale epidemiological surveys. These methods differentiate between trypanosome species and subspecies using specific primers and have been adapted to be able to simultaneously detect multiple species [25]. The internal transcribed spacer (ITS) region of ribosomal DNA offers a good target for universal trypanosome tests with highly conserved flanking regions, size variability among trypanosome species and subspecies and high copy number of around 200 [25]. Kinetoplastid (KIN) primers amplify the ITS1 [23, 25]. Sensitivity and specificity of the KIN-PCR is high for most of the pathogenic trypanosomes, but exhibit low sensitivity for detection of east African *T. vivax* [25] which was subsequently improved [26]. In order to increase the sensitivity and specificity, Cox et al. [27] used nested ITS-PCR. Adams et al. [28] have employed ITS-PCR techniques for trypanosome species differentiation in tsetse flies using fluorescent labelling. By means of ITS-PCR and multiple samples taken from single FTA cards, Cox et al. [29] demonstrated that a single punch from an FTA card is not sufficient to confirm the infectivity status of an individual animal as parasite DNA is unevenly distributed across the card. Furthermore, Ahmed et al. [30] have recently demonstrated that the use of an elution step simultaneously from 10 punches using Chelex 100® shows higher sensitivity compared to PCR of multiple punches separately. Ahmed et al. [31] further modified the ITS-PCR designed by Cox et al. [27] to include a Chelex 100® elution stage for detection of trypanosome species in cattle samples. Thus the KIN and the ITS1 primers, both targeting the ITS1 of rDNA, offer promise in the routine diagnosis of pathogenic trypanosomes in clinical specimens from infected animals.

LAMP is a novel strategy which amplifies DNA with high sensitivity and rapidity under isothermal conditions (60-65 °C), producing large quantities of DNA within an hour [24]. LAMP has the advantage over PCR of being cheaper and user-friendly and the LAMP products can be visualized by naked eyes or through measurement of turbidity or fluorescence [22, 32].

The present study, conducted in Zambia’s Northern, Muchinga and Eastern provinces where both AAT and HAT are endemic, combined microscopy, PCR and LAMP to examine the presence of different trypanosome species in cattle, goats and tsetse flies.

## Methods

### Study area and study design

**Fig. 1:**
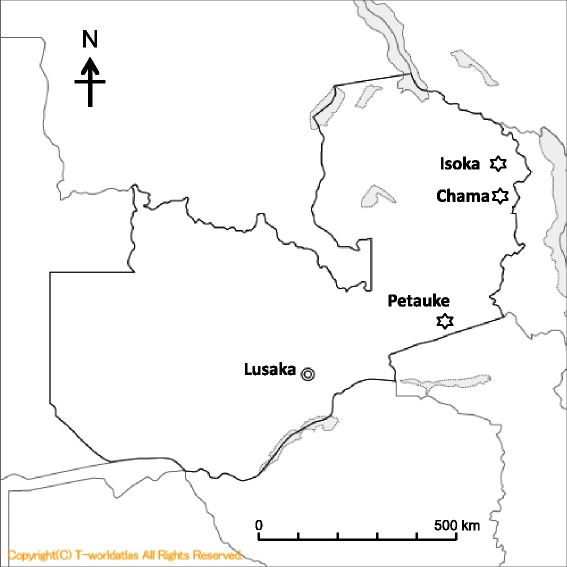
Map of Zambia showing location of Isoka, Chama and Petauke districts

Studies were conducted between May and October in 2008 and 2010 in Petauke (Eastern Province), Chama (Muchinga Province) and Isoka (Northern Province) districts, north-eastern Zambia (Fig. 1). A total of 243 indigenous and mixed breeds of cattle from Isoka (92), Petauke (46) and Chama (105) and 36 indigenous breeds of goats (all from Chama), were examined for presence of trypanosome species using microscopy, PCR and LAMP assays. In addition, PCR and LAMP assays were used to examine the presence of trypanosome species in 546 tsetse samples (caught from Mbambanda Zaro sanctuary, Chama) comprising 492 *Glossina m. morsitans* (27 males and 465 females) and 54 *G. pallidipes* (all males). Mbambanda Zaro sanctuary, lying on the border with Malawi, is part of the Zambia wildlife authority (ZAWA). It has abundant wildlife and tsetse flies and is surrounded by human settlements that maintain limited livestock, mainly goats.

### Sample collection and DNA preparation

Blood was collected from the jugular vein of cattle and goats whose owners consented to participate in the survey. Using vaccutainer tubes with EDTA-2Na (Terumo, Japan), 5 ml of blood was drawn from each animal, loaded into capillary tubes, packed cell volume (PCV) values determined and the buffy coat immediately examined for the presence of trypanosomes under light microscope [33]. In addition, total DNA of each blood sample (1 ml) was isolated using DNA Isolation Kit for Mammalian Blood (Roche Diagnostics K.K., Japan), following the manufacturer’s suggested protocol. The resultant DNA was stored at minus 30 °C until use.

Tsetse flies were trapped from Mbambanda Zaro sanctuary, Chama district, using baited epsilon trap which were set in tsetse fly favorable biotopes (mainly in shrubs adjacent to roads and footpaths within the sanctuary, and adjacent to homesteads in the immediate vicinity of the sanctuary). The traps, typically deployed at a distance of 100 m apart in a shade of a tree to reduce fly mortality due to heat, were emptied twice a day during 3 days of capture at Mbambanda Zaro sanctuary. The caught tsetse flies were separated according to sex and species and stored in 1.5 ml tubes containing silica gel. After grinding the dried tsetse flies using a disposable homogenizer (BioMasher I, Polysciences, Inc. PA. USA), total DNA of each tsetse fly was isolated using specific DNA isolation kit for Cells and Tissues (Roche Diagnostics, Germany) and stored at minus 30 °C until use.

### Detection of trypanosome species by PCR and LAMP

This study applied 3 different PCR techniques to detect trypanosome DNA in cattle, goats and tsetse flies: (i) KIN-PCR that amplifies the internal transcribed spacer 1 (ITS-1) of African trypanosomes [25], (ii) TviCatL-PCR that amplifies the highly conserved Cathepsin L-like gene among *T. vivax* isolates [34] and (iii) SRA-PCR that specifically amplifies the human serum resistance-associated (SRA) gene uniquely expressed by *T. b. rhodesiense* [6]. In addition, we also used PFR-LAMP that amplifies the paraflagella rod A gene of *Trypanozoon* subgenus (*T. b. brucei*, *T. b. gambiense*, *T. b. rhodesiense*, *T. evansi*, *T. equiperdum*) [35].

The reaction mixtures of LAMP (25 μl) and PCR (50 μl) and their respective amplification conditions were previously described [23, 25, 36]. Genomic DNA for each trypanosome species was used as positive control in the corresponding species-specific PCR or LAMP while *T. b. rhodesiense* (IL1501) and *T. congolense* Savannah (IL3000) DNA were used as positive controls in the KIN-PCR. In the case of KIN-PCR, the results were positive when the specific size product was observed [25], while in the case of LAMP, positive samples exhibited a bright fluorescent green colour when visualized under the transilluminator [12]. Double distilled water (DDW) was used as negative control. All samples detected as positive for *Trypanozoon* by either KIN-PCR or PFR-LAMP assays were screened for *T. b. rhodesiense* using SRA-PCR.

### Statistical analysis

Mean PCV values in trypanosome infected and non-infected livestock were compared using the student *t*-test. P values <0.05 were considered statistically significant. The kappa coefficient (K) was used to determine the agreement between two diagnostic tests. Calculations and interpretations of K values followed the methods of Viera and Garrett [37], with a value of 1 indicating perfect agreement and 0 indicating agreement equivalent to chance.

## Ethical clearance

This study received approval from the Provincial and District Veterinary Officers in the respective provinces and districts and from ZAWA. Informed consent was sought from livestock owners to participate in the survey and collect blood from their animals.

## Results

### Detection of trypanosome species in cattle and goat blood

Trypanosome infections detected in cattle and goats by microscopy, PCR and LAMP are summarized in Table 1. The overall proportion of animals (cattle and goats) infected with AAT was 6.1 % (95 % CI: 3.3-8.9 %) by microscopy, 7.5 % (95 % CI: 4.4–10.6 %) by PCR and 18.6 % (95 % CI: 13.6–23.6 %) by PFR-LAMP. PRF-LAMP was more sensitive for detecting *Trypanozoon* (~17 times) than KIN-PCR. Surprisingly, *Trypanosoma theileri* was parasitologically detected in one cow in Chama which tested negative for both KIN-PCR and PRF-LAMP. Although a number of parasitologically AAT positive animals were also positive for PCR and/or LAMP, there was poor agreement between Microscopy and both PCR (Kappa = 0.26) and LAMP (Kappa = 0.04).

**Table 1:** Detection of trypanosome infections in cattle and goats from Petauke and Chama districts by microscopy PCR and LAMP

District	Animals			SRA-PCR		KIN-PCR				TviCatL-PCR		PFR-LAMP	
						*Trypanozoon*		*T. congolense*					
				+	-	+	-	+	-	+	-	+	-
Petauke	Cattle	Microscopy	+	0	6	1^a^	5	0	6	1	5	3	3
			+										
			_-_	0	40	0	40	8	32	3	37	12	28
Isoka	Cattle		+	0	6	0	6	0	6	0	6	0	6
			_-_	0	86	0	86	0	86	0	86	5	81
Chama	Cattle		+	0	3	0	3	1	2	1^c^	2	1	2
			-	1^b^	101	1^b^	101	0	102	0	102	27	75
	Goats		+	0	2	1	1	0	2	1	1	1	1
			-	0	34	0	34	3	31	1	33	3	31

^a^This cow was also PFR-LAMP positive. ^b^This was the same cow and also PFR-LAMP positive. ^c^This cow was also diagnosed with *T. vivax* by microscopy based on morphology and rapid forward movement

Petauke district recorded the highest proportion of animals infected with AAT, mainly caused by *Trypanozoon* (32.6 %, 95 % CI: 18.6–46.6 %, by PFR-LAMP), *T. congolense* (savannah type mainly) (17.4 %, 95 % CI: 6.4–28.4 %, by KIN-PCR) and *T. vivax* (8.7 %, 95 % CI: 4.7–12.5 %, by TviCatL-PCR) while the lowest proportion was recorded in Isoka (5.4 %, 95 % CI: 0.8–10.0 %, by PFR-LAMP) exclusively caused by *Trypanozoon* (Table 1). Of those, 8.7 % (95 % CI: 5.6–11.8 %) and 0.7 % (95 % CI: 0.4-1.0 %) were mixed infections of *Trypanozoon* (PFR-LAMP) and *T. congolense* (KIN-PCR) in Petauke and Chama, respectively, while 2.2 % (95 % CI: 1.1–3.3 %) were mixed infections of *Trypanozoon* (PFR-LAMP), *T. congolense* (KIN-PCR) and *T. vivax* (TviCatL-PCR) in Petauke.

Data obtained from Chama district suggest that the overall occurrence of AAT was similar in cattle (28.6 %, 95 % CI: 20.0–37.4 %) and goats (27.8 %, 95 % CI: 12.8–42.4 %) although cattle recorded more *Trypanozoon* infection (26.7 %, 95 % CI: 18.4–35.2 %, PFR-LAMP) than goats which recorded greater infection with *T. congolense* (8.3 %, 95 % CI: 3.0–13.6 %, by KIN-PCR) and *T. vivax* (5.5 %, 95 % CI: 1.3–9.5 %, by TviCatL-PCR). Zoonotic *T. b. rhodesiense* was only detected in cattle (0.95 %, 95 % CI: -1.1–2.8 %, SRA-LAMP) from Chama district (Table 1).

**Fig. 2:**
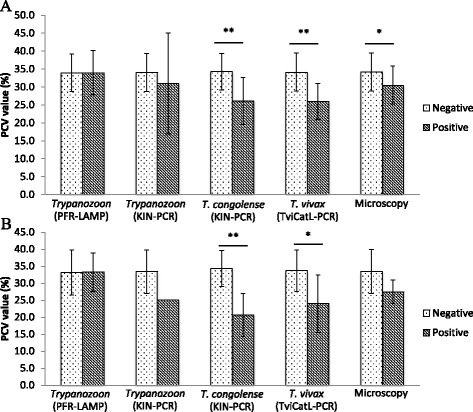
Comparison of PCV values between trypanosome infected and non-infected animals. **a** Cattle blood samples from all the 3 districts (*n =* 243). **b** Goat blood samples from Chama (*n =* 36). Values are mean ± SD. * Significant difference by Student’s *t*-test (*p < 0.05, **p < 0.001). Note: only 1 goat was detected to be trypanosome positive by KIN-PCR

Anaemia is one of the pathogenic consequences of infection with animal infective trypanosomes [5]. Because low PCV is one of the indicators of anaemia, we examined the association between infection with various trypanosome species and the level of PCV in such infected livestock. PCV values in cattle and goats ranged from 13.0 % to 50.0 % and 17.0 % to 46.0 %, respectively (Fig. 2). A significant reduction of PCV in animals with detectable parasitaemia of trypanosomes was observed in cattle (*p <* 0.05, Fig. 2a). Cattle and goats detected with *Trypanozoon* parasites by PFR-LAMP generally had similar PCV values to those of uninfected animals (*Trypanozoon* positive cattle: PCV = 35.0 ± 5.4 %; *Trypanozoon* negative cattle: PCV = 34.3 ± 4.9 %; *Trypanozoon* positive goat: PCV = 33.3 ± 5.7 %; *Trypanozoon* negative goat: PCV = 35.2 ± 3.3 %). In contrast, infection of cattle and goats (positive by either microscopy or PCR) with *T. congolense* (*p <* 0.001) or *T. vivax* (*p <* 0.05) was associated with significant reduction in PCV values (Fig. 2).

### Detection of trypanosome species in tsetse flies

The 546 tsetse flies from Mbambanda Zaro sanctuary, Chama district, were examined for the presence of trypanosome species by PCR and LAMP. Table 2 summarizes the obtained data. Trypanosome DNA was identified in 36.4 %, 95 % CI: 23.8–49.0 % (by PCR) and 40.1 %, 95 % CI: 25.0–55.2 % (by PFR-LAMP) of the tsetse flies, respectively. The main trypanosome DNA detected was *Trypanozoon* (30.4 %, 95 % CI: 19.8–41.0 %, by PFR-LAMP), *T. vivax* (25.1 %, 95 % CI: 15.6–34.6 %, by TviCatL-PCR) and *T. congolense* (mainly savannah type and to a lesser extent kilifi type) (11.2 %, 95 % CI: 4.9–17.5 %, by KIN-PCR). PFR-LAMP was more sensitive for detecting *Trypanozoon* (>21 times) than KIN-PCR. There was poor agreement between LAMP and KIN-PCR (Kappa = 0.38). *Trypanozoon* DNA was only detected in *G. m. morsitans*, with *T. b. rhodesiense* DNA being detected in 3.5 % (95 % CI: 1.9–5.1 %, by SRA-PCR) of the flies.

**Table 2:** Detections of trypanosome species in tsetse flies from Chama district by PCR and LAMP

Tsetse fly species			SRA-PCR		KIN-PCR				TviCatL-PCR	
					*Trypanozoon*		*T. congolense*			
			+	_-_	+	_-_	+	_-_	+	_-_
*G. m. morsitans*	PFR-LAMP	+	16	36	8^a^	44	10^b^	42	11^c^	41
(females)		_-_	0	413	0	413	45	368	94	319
*G. m. morsitans*		+	3^d^	0	1^e^	2	1^e^	2	3^d^	0
(males)		-	0	24	0	24	1	23	10	14
*G. pallidipes*		+	0	0	0	0	0	0	0	0
(males)		-	0	54	0	54	4^f^	50	19^g^	35

^a^Two samples were SRA-PCR positive. ^b^Six samples were positive for *Trypanozoon* by KIN-PCR and PFR-LAMP; 3 SRA- PCR positive; 1 TviCatL-PCR positive. ^c^Two samples were SRA-PCR positive. ^d^These were same samples. ^e^This was the same SRA-PCR and TviCatL-PCR positive sample. ^f^One sample tested positive for both *T. congolense* Savannah and Kilifi type. ^g^One sample also tested positive for *T. congolense*Savannah type

## Discussion

Diagnosis of AAT and HAT in endemic regions remains a big challenge. In the present study, several tests were applied to determine AAT infection in cattle and goats and the presence of trypanosome species in tsetse vectors. As expected, microscopy exhibited relatively lower sensitivity than PCR and LAMP. Of note, there was poor agreement among the test methods used for detection of trypanosomes species in animal blood and tsetse flies (Kappa <0.40). For instance, the failure of PCR and LAMP to detect and identify 6 parasitologically positive cattle samples in this study was unexpected. These discrepancies may be resolved by the use of more sensitive trypanosome-species-specific PCR and/or LAMP. Furthermore, *T. theileri* was only detected in one cow in Chama district by microscopy. Although cattle frequently habour *T. theileri* [27, 29, 31], the KIN-PCR was unable to detect that parasite in the present study. This may be a result of lower sensitivity of the test [26] and/or possible occurrence of inhibitors in cattle blood [35]. In agreement with the former notion, detection of *T. theileri* has mainly been reported when more sensitive ITS-PCR such as nested ITS-PCR [27] or modified nested ITS-PCR [31] were used.

The major advantage of molecular tests over microscopy is for epidemiological studies to identify trypanosome species. ITS-PCR can identify several pathogenic trypanosome species in a single PCR reaction, reducing on the cost of PCR diagnosis and allowing for a greater number of field samples to be tested in epidemiological studies [25, 26]. However, in the present study, in agreement with previous reports [25, 26], there was little *T. vivax* detected by KIN-PCR. *T. vivax* is very diverse, comprising 3 main groups including (i) East African, (ii) West African and (iii) South American isolates [34], and makes diagnosis difficult. TviCatL-PCR was required to complement on the low sensitivity of KIN-PCR for *T. vivax* detection [34]. Furthermore, this study confirmed that the KIN-PCR has significantly lower sensitivity for *Trypanozoon* detection than the PFR-LAMP [36].

In this study, some cattle were given curative treatments for AAT several days before sampling. Since longevity of trypanosome DNA after treatment remains to be established, we cannot rule out the possibility of the LAMP assay being better at picking up residual DNA from dead trypanosomes in cattle blood than PCR. All KIN-PCR positive samples were also PFR-LAMP positive, suggesting that the KIN-PCR may be recommended for epidemiological surveillance of *T. congolense* in AAT endemic regions, but it would not be appropriate for *T. vivax* or *Trypanozoon* surveillance. Finally, whereas both the KIN-PCR and the PFR-LAMP are unable to distinguish between the closely related *T. brucei* subspecies, SRA-PCR is needed to specifically distinguishing the human-infective *T. b. rhodesiense* from the animal-infective *T. b. brucei* [6].

Our data show that Petauke district (58.7 %) reported the highest occurrence of AAT, followed by Chama (28.6 %), with Isoka (5.4 %) having the lowest. Of note, the animals in all the districts were mostly infected with *Trypanozoon* parasites such as *T. b. brucei*. However, such *Trypanozoon*-infected animals either exhibited only subclinical symptoms of AAT or were asymptomatic altogether, suggesting that those trypanosomes do not have significant effect on the general animal health [3, 6]. In sharp contrast, infection with *T. congolense* (mainly Savannah type) and *T. vivax* seems to be associated with clinical AAT as evidenced by significantly lower PCV values. In particular, *T. congolense* strains within the Savannah type are regarded to be the most pathogenic/virulent and widespread throughout the savannah ecosystem of SSA [38]. Accordingly, Marcotty et al. [39] suggested that low PCV could be used as an indicator of infection with *T. congolense*. On the other hand, however, it is important to note that there were several anaemic animals that were trypanosome negative, suggesting that infection or co-infection with other haemoparasites or worms could also induce anaemia [40]. Interestingly, there was hardly any case of clinical AAT in Isoka district (recorded no case of *T. congolense* or *T. vivax* infection) and Chama district (recorded very low prevalence of both parasite species [~1.0 %]). According to the District Veterinary Officers, most of the sampled cattle in the two districts were treated with isometamidium chloride a few weeks prior to the sampling exercise. Our data is in agreement with previous reports that *T. congolense* is the major cause of clinical AAT in both cattle and goats [3–5].

The detection of the human-infective *T. brucei* subspecies in cattle in Chama district is a significant finding albeit the low prevalence (~1.0 %). This is in view of the increased cases of HAT that were being reported from Chama around the time of sampling [12]. According to recent reports from Uganda [6, 7], cattle have been implicated to be the principal domestic reservoirs of HAT. Cattle have also been previously documented as reservoirs of *T. b. rhodesiense* in Kenya [8] and Tanzania [9–11]. Furthermore, we also recently documented the detection of *T. b. rhodesiense* in dogs from Zambia’s Luangwa and Zambezi valley foci where HAT is re-emerging [17, 18]. Although we did not investigate the presence of trypanosome species in wildlife, several wild animals have previously been documented to act as reservoirs of *T.b. rhodesiense* in Zambia’s Luangwa valley ecosystem, including greater kudus (*Tragelaphus strepsiceros*), warthogs (*Phacochoerus africanus*), bushbuck (*Tragelaphus scriptus*), duiker (*Sylvicapra grimmia*), giraffe (*Girraffa camelopardalis*), impala (*Aepyceros melampus*), lion (*Panthera leo*), waterbuck (*Kobus ellipsiprymnus*), zebra (*Equus quagga boehmi*) and African buffalo (*Syncerus caffer*) [19, 41–43]. The evidence of detection of *T. b. rhodesiense* in *G. m. morsitans* in the present study is intriguing in view of the latter’s ability to take blood meals from multiple hosts including wildlife, domestic animals and humans (*Homo sapiens*) (mainly wildlife staff and hunters), facilitating the circulation of the parasite within the ecosystem. Dennis et al. [44] also recently detected *T. b. rhodesiense*, mainly in *G. m. morsitans* (35.3 %), and to a lesser extent in *G. pallidipes* (0.5 %), in their studies in Zambia’s Luambe National Park, within the Luangwa valley ecosystem. Taken together, these data suggest that *G. m. morsitans* flies are the main vectors of *T. b. rhodesiense* in the Luangwa valley ecosystem. It is, however, noteworthy that the actual prevalence of *T. b. rhodesiense* in both cattle blood and tsetse flies may be higher in view of the fact that SRA is a single copy gene which may be difficult to detect in low parasitaemias where there is not enough DNA to amplify it [6, 45]. Moreover, we may not establish from our assays whether we were detecting active infection in the tsetse flies or residual DNA from dead trypanosomes that the flies may have picked following a blood meal from treated animals.

In agreement with Anderson et al. [19], results obtained in the present study indicate that various trypanosome species circulate within a wide and diverse host community in the bio-diverse Luangwa valley ecosystem. The epidemiological stability of the diverse Luangwa valley ecosystem is being eroded by an influx of people and their livestock, encroaching into the Luangwa valley. Overall, these data should be used to trigger a “One Health” approach towards HAT control through disease intervention in livestock, wildlife and tsetse vectors [46].

## Conclusions

The present study employed several diagnostic tools to examine the presence of different trypanosome species in cattle, goats and tsetse flies. There was poor agreement among the test methods used for detection of trypanosomes species in animal blood and tsetse flies (Kappa <0.40). Microscopy generally exhibited relatively lower sensitivity than PCR and LAMP. Among the molecular tests, the KIN-PCR was found to be sensitive for the detection of *T. congolense* but not for *T. vivax* or *Trypanozoon* which were better detected by TviCatL-PCR and PFR-LAMP, respectively. Thus the KIN-PCR may be recommended in epidemiological surveillance of *T. congolense* in AAT endemic regions, while species-specific PCR and/or LAMP might be of greater value in the detection of *T. vivax* or *Trypanozoon*, including human-infective *T. b. rhodesiense*.

## Abbreviations

AAT: Animal African trypanosomiasis
DDW: Double distilled water
HAT: Human African trypanosomiasis
SRA: Serum resistance-associated
ITS: internal transcribed spacers of the ribosomal DNA
KIN: primers that target the internal transcribed spacer region of kinetoplastid ribosomal DNA
LAMP: Loop-mediated isothermal amplification
PCR: Polymerase chain
PCV: Packed cell volume reaction
PFR: Paraflagella rod A gene
SRA: Human serum resistance-associated antigen
