# Erratum to: Studies of trypanosomiasis in the Luangwa valley, north-eastern Zambia

**DOI:** 10.1186/s13071-015-1173-y

**Published:** 2015-10-22

**Authors:** Dusit Laohasinnarong, Yasuyuki Goto, Masahito Asada, Ryo Nakao, Kyoko Hayashida, Kiichi Kajino, Shin-ichiro Kawazu, Chihiro Sugimoto, Noboru Inoue, Boniface Namangala

**Affiliations:** O.I.E. Reference Laboratory on Surra, National Research Center for Protozoan Diseases, Obihiro University of Agriculture and Veterinary Medicine, Inada-cho, Obihiro, 080-8555 Hokkaido Japan; Clinical Sciences and Public Health Department, Faculty of Veterinary Science, Mahidol University, 999 Phuttamonthon 4 Road, Salaya, Phuttamonthon, Nakhon Pathom 73170 Thailand; Laboratory of Molecular Immunology, Graduate School of Agricultural and Life Sciences, The University of Tokyo, Bunkyo-ku, 113-8657 Tokyo Japan; Research Center for Zoonosis Control, Hokkaido University, Sapporo, 060-0818 Hokkaido Japan; Department of Paraclinical Studies, School of Veterinary Medicine, University of Zambia, Lusaka, P.O. Box 32379 Zambia

Unfortunately, the original version of this article [1] contained a mistake. The spelling of Yasuyuki Goto’s name was incorrectly given as Yasuhuki Goto. The correct spelling is Yasuyuki Goto and is included correctly in the author list of this article.

In addition, some author affiliations were assigned incorrectly. Yasuyuki Goto additionally belongs to affiliation 3, which should be listed as ‘Laboratory of Molecular Immunology’ and not ‘Department of Molecular Immunology’. Masahito Asada was incorrectly assigned to affiliation number 3, but belongs to affiliation 1. This has been corrected in the affiliation list of this article.
